# Severe Human Bocavirus Infection, Germany

**DOI:** 10.3201/eid1712.110574

**Published:** 2011-12

**Authors:** Robert Walter Körner, Maria Söderlund-Venermo, Silke van Koningsbruggen-Rietschel, Rolf Kaiser, Monika Malecki, Oliver Schildgen

**Affiliations:** Children’s Hospital of the University of Cologne, Cologne, Germany (R.W. Körner, S. van Koningsbruggen-Rietschel);; University of Helsinki and Haartman Institute, Helsinki, Finland (M. Söderlund-Venermo);; University Hospital of Cologne, Cologne (R. Kaiser);; Kliniken der Stadt Köln gGmbH, Cologne (M. Malecki, O. Schildgen)

**Keywords:** human bocavirus, infection, pneumonia, PCR, serology, infant, viruses, Germany

## Abstract

Human bocavirus (HBoV), discovered in 2005, can cause respiratory disease or no symptoms at all. We confirmed HBoV infection in an 8-month-old girl with hypoxia, respiratory distress, wheezing, cough, and fever. This case demonstrates that lower respiratory tract infection caused by HBoV can lead to severe and life-threatening disease.

Human bocavirus (HBoV; family *Parvoviridae*; genus *Bocavirus*) was discovered in 2005 and is distributed worldwide (*1*). Four species of HBoV have been identified (HBoV1–4). Increasing evidence indicates that HBoV causes infections of the respiratory tract. Numerous studies depict HBoV as a co-pathogen but also show that its prevalence in asymptomatic patients is high.

HBoV has gained considerable clinical relevance since its discovery. It has been detected in respiratory specimens, and when it causes disease, it seems to have a broad spectrum of signs and symptoms. Although certain features make HBoV infection distinguishable from other viral infections, a diagnosis cannot be made clinically. We report a case of severe HBoV infection that led to hypoxia, respiratory distress, wheezing, cough, and fever.

## The Patient

On January 2, 2011, an 8-month-old girl with acute respiratory distress was seen in the emergency department of the Children’s Hospital of the University of Cologne. The girl’s parents had noticed labored breathing, intercostal retractions, and fever for 1 day. Further medical history was unremarkable. No one else who had contact with the girl was sick.

At admission, the girl was lethargic and inappetent with a fever of 38.3°C and oxygen saturation of 86%. Expiration was prolonged, and an expiratory wheeze was heard. Breath sounds over the right upper lung lobe were remarkably diminished. The patient had submandibular lymphadenopathy and mild pharyngitis but no rash or abnormalities of the heart or abdomen. The clinical signs were typical of an upper respiratory tract infection combined with severe obstructive bronchitis.

Chest radiographs showed diffuse bilateral infiltrates and total atelectasis of the right upper lung lobe. Blood analysis indicated partial CO_2_ concentration within normal limits and slightly elevated C-reactive protein level (10 mg/L). Electrolytes and renal and hepatic markers were within reference ranges, as was complete blood count except for thrombocytosis (744,000 platelets/µL).

Treatment with prednisolone and inhalation therapy delivering albuterol and ipratropium by vaporizor was initiated. Because of the lung atelectasis, the child received cefuroxime as prophylaxis for 2 weeks. Oxygen saturation could be restored to within reference limits with nasal cannula oxygen delivery at 4 L/min.

During the next 2 days, the patient’s body temperature was 38.0°C–39.0°C, and she remained lethargic. A nasopharyngeal aspirate was obtained 4 days after symptom onset and screened for respiratory viruses by real-time PCR (*2*) and Luminex xTAG RVP panel (Abbott, Wiesbaden, Germany). Each assay had positive results for HBoV DNA and negative results for influenza A virus; influenza A virus subtype H1N1; influenza B virus; parainfluenza viruses types 1–4; adenovirus; human metapneumovirus; coronaviruses NL63, HKU1, 229E, and OC43; respiratory syncytial virus (RSV); rhinovirus; and enterovirus. The aspirate was not tested for bacteria. Cultures of blood collected on the day of admission, before antimicrobial drug treatment was started, were negative. A second chest radiograph showed partial atelectasis of the left upper lung lobe, a retrocardial infiltrate, and pneumomediastinum. On hospitalization day 5, oxygen saturation could be maintained at a lower flow rate. On day 6, chest radiograph showed regression of atelectasis in both lobes and of the pneumomediastinum. Temperature dropped to subfebrile levels, and the child’s general condition was improving. On day 9, no additional oxygen was needed. On day 10, chest radiograph showed residual atelectasis in both upper lobes and residual infiltrates in both lungs, and the patient was discharged. Two weeks later, at follow-up examination, the child was doing well, breath sounds were unremarkable, and the pathologic radiographic findings had resolved completely.

To verify that the disease was caused by an HBoV infection, we performed a real-time PCR targeting the nonstructural protein 1 region of HBoV; we detected 5,819 copies/mL HBoV DNA in the patient’s serum 30 days after symptom onset (*3*). The PCR was conducted with primers OS1 and OS2 as described (*4*), and an HBoV subtype 2 plasmid dilution series (kindly provided by Tobias Allander, Karolinska Institute, Stockholm, Sweden) was used as a quantitative control. Sequencing was performed by an external sequencing service (Eurofins MWG, Munich, Germany); the same primers from both directions were used.

Sequencing and BLAST analysis (http://blast.ncbi.nlm.nih.gov/Blast.cgi) confirmed the virus to be HBoV1, not HBoV2–4. Additionally, HBoV-specific IgM and IgG and avidity of IgG were measured by sensitive and specific enzyme immunoassays (*5*,*6*). Results were clearly positive for both: IgM absorbance 0.385 and cutoff 0.167; IgG absorbance 2.971 and cutoff 0.188. IgG was of low avidity (4%, cutoff 15%), indicating acute primary HBoV infection. Control samples were tested as described (*5*–*8*).

## Conclusions

HBoV has been detected worldwide in nasopharyngeal aspirates collected for screening (*9*). Large-scale studies showed that HBoV can be detected in children with signs and symptoms of respiratory tract infection. Studies have detected HBoV in 9% and 19.3% of all samples, indicating that it is the second or third most commonly detected virus, after RSV and rhinovirus (*2*,*10*). Thus, the overall contribution of HBoV to all detected respiratory viruses lies well below that of RSV. High prevalence does not necessarily mean high clinical relevance, and proving its causative role has been difficult because the virus is often detected along with other viruses; co-detection rates are as high as 75% (*11*,*12*).

HBoV can also persist for months in the respiratory tract after resolution of disease, further complicating diagnosis (*12*), which therefore should be based on detection of HBoV DNA in blood and measurement of HBoV-specific antibodies. HBoV is the most probable cause of respiratory tract disease if the patient has a high viral load in nasopharyngeal aspirates accompanied by viremia, if HBoV is the only pathogen detected, and if an acute primary HBoV infection is diagnosed by serologic testing (*5*,*11*,*13*). We detected HBoV DNA in nasopharyngeal aspirate and serum. The serologic results strongly suggest that this child had an acute primary HBoV infection. The serologic assay developed earlier was validated in a series of studies and shown to reliably measure serologic response against HBoV (*5*–*8*). Inflammatory markers were not elevated, thereby indicating no bacterial infection. However, the contribution of bacteria to the course of the disease cannot be completely ruled out.

Signs and symptoms for the patient reported here have been described as the most common signs and symptoms of HBoV-infected children (*14*) and are typical of lower respiratory tract viral infections in general. In 1 study (*14*), 18 patients had an HBoV single infection and 2 had lobar atelectasis, as did the patient reported here. Similarly, another case report described HBoV infection that resulted in pneumomediastinum (*15*). The age of the patient reported here is also typical of HBoV, which mostly infects children 6 months to 3 years of age, but it does not usually infect young infants, as RSV does (*14*). The case reported here shows that a lower respiratory tract infection caused by HBoV can lead to severe and life-threatening disease.
